# Sedentary Behavior and Physical Function Decline in Older Women: Findings from the Women's Health Initiative

**DOI:** 10.1155/2012/271589

**Published:** 2012-05-21

**Authors:** Rebecca Seguin, Michael LaMonte, Lesley Tinker, Jingmin Liu, Nancy Woods, Yvonne L. Michael, Cheryl Bushnell, Andrea Z. LaCroix

**Affiliations:** ^1^Fred Hutchinson Cancer Research Center, Seattle, WA 98109, USA; ^2^Group Health Research Institute, Seattle, WA 98101, USA; ^3^Department of Social and Preventive Medicine, University at Buffalo, Buffalo, NY 14214, USA; ^4^School of Nursing, University of Washington, Seattle, WA 98195, USA; ^5^Drexel University School of Public Health, Philadelphia, PA 19102, USA; ^6^Department of Neurology, Wake Forest University Health Sciences, Winston Salem, NC 27157, USA

## Abstract

Sedentary behavior is associated with deleterious health outcomes. This study evaluated the association between sedentary time and physical function among postmenopausal women in the Women's Health Initiative Observational Study. Data for this prospective cohort study were collected between 1993–1998 (enrollment) and 2009, with an average of 12.3 follow-up years. Analyses included 61,609 women (aged 50–79 years at baseline). Sedentary time was estimated by questionnaire; physical function was measured using the RAND SF-36 physical function scale. Mixed-model analysis of repeated measures was used to estimate the relationship of sedentary time exposures and changes in physical function adjusting for relevant covariates. Compared to women reporting sedentary time of ≤6 hours/day, those with greater amounts of sedentary time (>6–8 hours/day, >8–11 hours/day, >11 hours/day) reported lower physical function between baseline and follow up (coefficient = −0.78, CI = −0.98, −0.57, −1.48, CI = −1.71, −1.25, −3.13, and CI = −3.36, −2.89, respectively *P* < 0.001). Sedentary time was strongly associated with diminished physical function and most pronounced among older women and those reporting the greatest sedentary time. Maintaining physical function with age may be improved by pairing messages to limit sedentary activities with those promoting recommended levels of physical activity.

## 1. Introduction

Participation in regular physical activity confers many health benefits, including reduced risk of heart disease, hypertension, stroke, dyslipidemia, obesity, diabetes, osteoporosis, certain cancers, and all-cause mortality [1–7]. Physical activity also improves skeletal muscle function and may ameliorate symptoms of arthritis, depression, and sleep disturbances in older women [8–11]. In general, greater quantity and intensity of exercise have increased benefits, although studies have demonstrated benefits with less-frequent, lower-intensity activities over short bouts [6]. Consistent with this evidence, prolonged exposure to sedentary time has been associated with increased risk of many of the aforementioned diseases and conditions [12–14].

In much of the research to date, sedentary time is classified based on lack of self-reported physical activity, as compared to asking about or measuring actual time spent sitting or lying. Specifically, an assessment of sedentary time may include time spent in behaviors such as screen viewing (i.e., watching television or working at a computer) and other daily episodes of sitting or lying to work, eat, read, or socialize. Studies examining associations of sedentary time with measures of general health or with risk of disability and disease are growing but still quite limited. Of those available, greater sedentary time has been shown to impart deleterious physiologic effects [15] and increased risk for obesity, metabolic syndrome, and type 2 diabetes [16–18]. Additionally, research from the Canada Fitness Survey examined data from 17,013 men and women, in which they observed a dose-response relationship between sitting time and mortality from all causes and cardiovascular disease after controlling for usual physical activity habits [19].

A related area of investigation is to understand the effects of sedentary time on physical function (PF)—including strength, mobility, and self-care—in aging populations. A recent report by Buman and colleagues examined objective accelerometer measures of physical activity in older adults and found that even low intensity physical activity was associated with health benefits, including greater PF [20]. While research has established the importance of regular physical activity participation for maintaining PF in older adults [8, 21], more data are needed to understand whether exposure to greater sedentary time after menopause is an independent risk factor for decline in PF at later ages, and to what extent other factors may influence this association. This study utilized data from the Women's Health Initiative Observational Study and Extension Studies (WHI-OSES) to examine these relationships in a well-characterized cohort of older postmenopausal women.

## 2. Methods

### 2.1. Purpose

This study aims to understand the relationship between sedentary time and decline in self-reported physical function among women in the WHI-OSES. We hypothesized that greater amounts of daily sedentary time would be associated prospectively with lower physical function scores. 

### 2.2. Study Population

The Women's Health Initiative (WHI) includes observational and randomized controlled intervention studies conducted among postmenopausal women at 40 clinical centers across the United States. The design and population for those studies have been previously described [22–24]. This study focuses on participants from the Women's Health Initiative Observational Study (WHI-OS) who continued with Women's Health Initiative Extension Study, herein WHI-OSES. Briefly, the WHI-OS with continued follow up in the WHI-OSES is a large prospective study conducted to investigate morbidity and mortality in aging women. The WHI-OS participants were recruited from 1993–1998 and included 93,676 racially and ethnically diverse women aged 50–79 years. Data from participants in WHI-OS were collected at baseline and follow up using in-person interviews, physical measurements, blood samples, and self-report questionnaires. 

The WHI Extension Study (WHI-ES) was designed to collect an additional five years of follow up data (2005–2010) among all women in WHI, including the randomized trial participants, to describe the long-term effects of the interventions as well as continue to optimize the vast scientific assets of the WHI-OS longitudinal dataset. Eligible WHI-OS participants were mailed information about the extension study at closeout of the original parent study (2004-2005) and asked to consider providing written consent for extended follow up and to update their personal contact information. 

Among the 93,676 WHI-OS women, 86,744 (92.6%) were eligible for the WHI-ES (women deemed ineligible were deceased, *N* = 5, 463 or had lost contact, *N* = 1, 469). Of the eligible women, 63,231 (72.9%) consented and were enrolled. Of these 63,231 WHI-OSES women, 61,609 provided data on baseline sedentary time exposure and PF required for these analyses. 

### 2.3. Physical Function

Physical function was assessed at baseline (1993–1998), year 3 (1996–2001), and each year of the WHI-ES (ES years 1–5; 2005–2010) using the ten PF items from the RAND SF-36 scale, a well-validated measure of self-reported PF [25, 26]. The PF scale includes items that measure whether current health limits PF in four general domains (moderate/vigorous activities (2 items), strength (4 items), walking abilities (3 items), and self-care (1 item)). Scoring is from 0–100; a higher score indicates superior PF. 

### 2.4. Sedentary Time

Sedentary time at baseline and at Year 3 was assessed using questions that asked the respondent to determine total hours spent sitting (e.g., at work, eating, driving, riding, watching TV, talking) in a usual given day, and the total hours spent lying down in a usual typical day (e.g., sleeping, trying to sleep, watching TV, resting, napping). Total sedentary time is calculated by combining the reported hours of sitting and lying time and then subtracting reported hours sleeping (asked separately from the question on lying time). 

### 2.5. Covariates

Additional baseline covariates of interest included age, race/ethnicity, income, education, body mass index (BMI, kg/m^2^), physical activities, self-reported health status, number of chronic diseases, smoking, alcohol use, hypertension, treated diabetes, history of stroke, history of coronary heart disease (CHD), history of congestive heart failure (CHF), history of chronic obstructive pulmonary disease (COPD), history of falls, history of cancer, history of arthritis, hormone use, living alone, number of falls in past 12 months, depressed mood, and activity of daily living disability. These variables were chosen due to their potential relationship with PF decline. In addition, models were adjusted for usual recreational physical activity. Physical activity was assessed by a questionnaire that captured duration and frequency of walking and other recreational activities according to intensity groups (mild, moderate, strenuous/very hard). Weekly energy expenditure (MET-hours/week) was computed by summing the product of duration and intensity for each physical activity, with intensity defined in metabolic equivalents (METs) obtained from a standardized classification system [27].

### 2.6. Statistical Approach

Baseline descriptive characteristics were examined by quartiles of sitting and total sedentary time. Since sample characteristics were similar according to sitting time and sedentary time, we present these data only by total sedentary time in Table 1. Two-sided *P*-values comparing these baseline characteristics across quartiles were based on Chi-Square tests. Values for sitting time and total sedentary time at baseline and year 3 along with change between those time points, stratified by age, are examined to determine the amount of change in these behaviors. 

Linear mixed model analysis of repeated measures was used to estimate mean PF scores across follow-up time in relation to sedentary time exposures, adjusted for the baseline covariates mentioned above. This method first attempted to estimate the covariance structure of PF scores across various follow up times by using both graphical tools and the information criteria statistics. The first-order autoregressive [AR (1)] covariance structure was chosen to best represent the correlation structure. Then, the mixed model assessed the relationship between sedentary time exposures and change in PF scores using generalized least squares with the estimated covariance. To fully utilize the data source, the model updated the sedentary time exposures at Year 3 when available to incorporate the change in exposure variables. 

To assess to what degree the association between sedentary behavior and decline in mean PF scores was affected by physical activity level, the interaction between baseline sedentary time and baseline physical activity as well as the interaction between year 3 sedentary time and year 3 physical activity on PF scores were evaluated in separate models, adjusting for the aforementioned set of covariates. In addition, to account for new onset of comorbidity (CHD, CHF, cancer, stroke, diabetes, hypertensive, and hip fracture over age 55) that occurred after year 3, secondary analyses repeated the primary analyses with adjustment for new onset of comorbidity. All statistical analyses were performed using SAS statistical software (version 9.2; SAS Institute Inc, Cary, NC, USA).

## 3. Results

Participant characteristics by quartile of total sedentary time (≤6, 6–8, 8–11, >11 hours/day) at baseline are shown in Table 1. Women within the first three quartiles were similarly distributed across age groups, with a modestly smaller percentage of 70–79-year olds in the fourth quartile. Women from minority race/ethnic groups were more likely to report the least sedentary time. Trends in education and income also showed higher percentages of the most highly educated and those with more income having greater amounts of the sedentary time. Women reporting less sedentary time reported higher physical activity levels and higher self-reported health status. They also had lower prevalence of obesity (BMI ≥ 30) and reported less-frequent depressed mood. Women with higher sedentary time reported a greater number of chronic diseases overall, although there were no differences in the prevalence of many specific diseases examined across quartiles of sedentary time. Women with greater sedentary time also reported a higher frequency of falls over the past 12 months, activity of daily living (ADL) disability, and were more likely to live alone. 

 Reported sedentary time at baseline and at year 3 were moderately correlated (Pearson *r* = 0.55). On average, women reported about a half-hour less of sitting time and total sedentary time at year 3 compared to baseline (Table 2). The greatest reductions occurred among the 50–59-year olds (−0.60 hours) and 60–69-year olds (−0.57 hours). This equates, approximately, to a reduction of 35 minutes of sedentary time per day among the 50–59- and 60–69-year olds and to a reduction of 20 minutes per day among the 70–79-year olds. 

After adjustment for covariates, greater amounts of sitting time and total sedentary time were significantly associated with lower mean PF scores (Table 3). Compared to those who reported sedentary time of ≤ 6 hours/day, those with greater amounts of sedentary time (>6–8 hours/day, >8–11 hours/day, and >11 hours/day) had lower PF scores between baseline and approximately 12 years of follow up [coefficient = −0.78, 95% confidence interval (CI) = −0.98, −0.57, −1.48, CI = −1.71, −1.25, −3.13, CI = −3.36, −2.89, resp.; *P* < 0.001]. Similar associations were seen across quartiles of sitting time and PF scores at follow up (coefficient = −0.96, CI = −1.16, −0.76; −1.45, CI = −1.68, −1.22, −2.45, CI = −2.69, −2.22, resp.; *P* < 0.001). Thus, in examining the relationships for total sedentary time, women reporting >11 hours per day had more than three times lower mean PF scores compared to women in the lowest category (≤6 hours per day), with comparable findings for sitting time, as shown in Table 3. The secondary analyses that accounted for new onset of comorbidity after year 3 revealed similar relationships (data not shown). 

**Figure 1:**
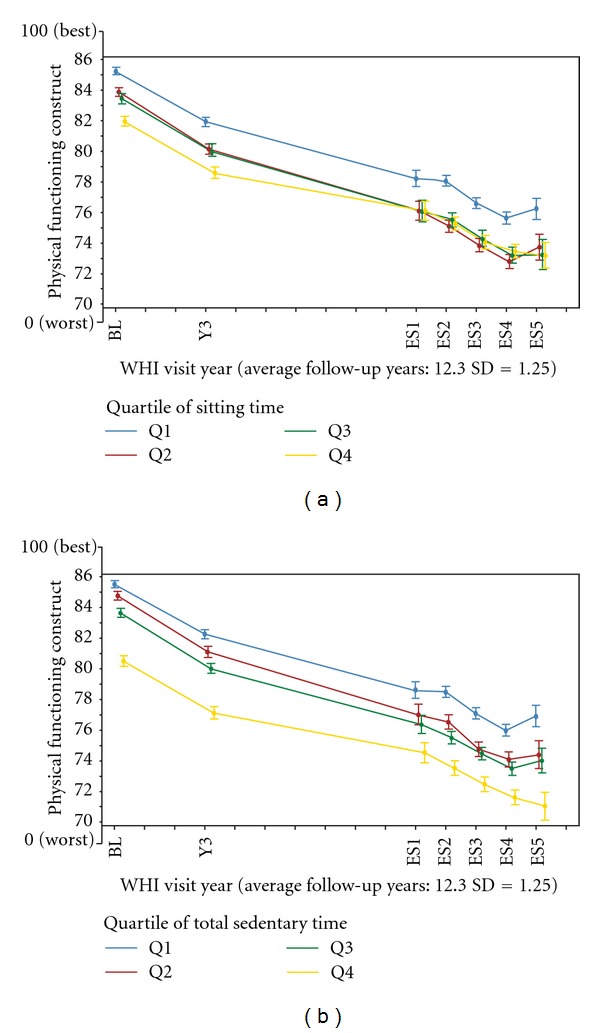
(a) Physical function score over time by baseline sitting time, *N* = 61,609. (b) Physical function score over time by baseline total sedentary time, *N* = 61,609.

**Figure 2:**
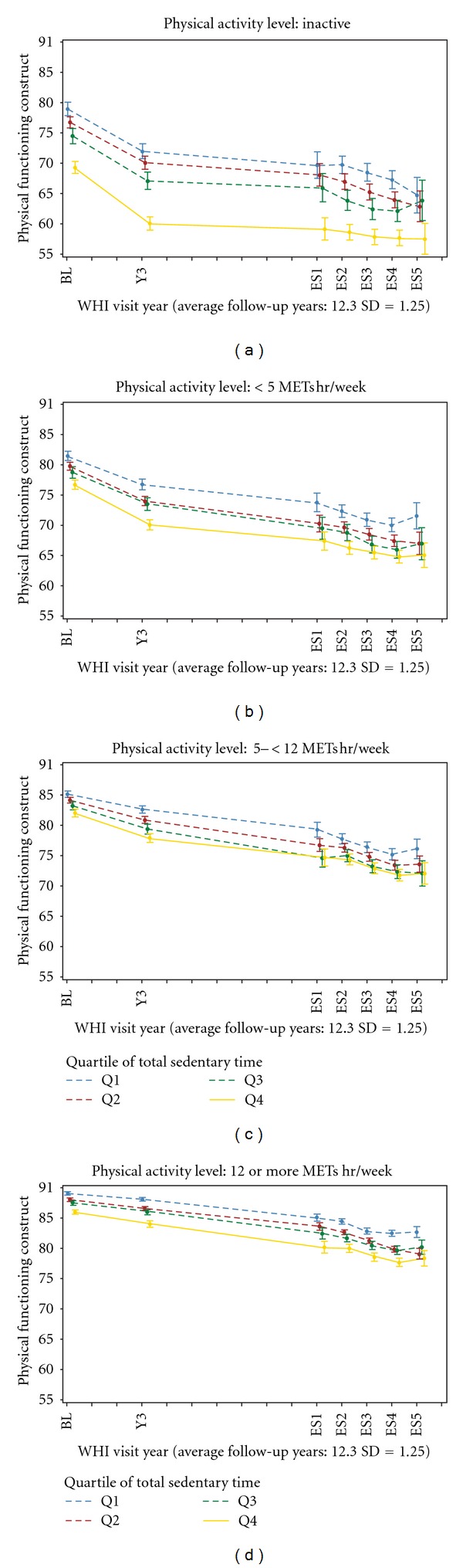
Physical function score over time by total sedentary time (year 3) and physical activity level, *N* = 61,609.

The interaction between baseline sedentary time and baseline physical activities on PF scores was borderline significant (interaction *P* = 0.0575). However, the association of year 3 sedentary time with change in mean PF scores differed by level of physical activity at year 3 (interaction *P* < 0.0001). Compared to women who were inactive and reported sedentary time of ≤5 hours/day at year 3, women who reported higher levels of physical activity and the same amount of sedentary time (≤5 hours/day) experienced less decline in PF. Figures 1(a) and 1(b) display physical function score over time by baseline sitting time and baseline total sedentary time, respectively. Figures 2(a)–2(d) show sedentary time quartiles across four levels of physical activity. 

## 4. Discussion

This paper contributes to a growing body of literature that supports the notion that sedentary time behavior may well be a distinct component of physical inactivity not merely reflecting an adverse exposure at the extreme low end of the physical activity continuum. We found that women reporting the largest amounts of sedentary time had higher PF scores compared to those reporting less sedentary time. This association was dose-responsive and independent of numerous potential confounding variables including self-reported physical activity from vigorous, moderate, and mild activities as well as walking. 

In addition, these findings not only demonstrate independent associations between total sedentary time and physical activity, but also show an interaction between the two activity variables. Greater amounts of physical activity were clearly related to higher PF scores. Nonetheless, in every category of physical activity, declines in PF were greatest in the women reporting the most sedentary time. Women with the least physical activity and the most sedentary time had the most precipitous drops in PF. Thus, even in the context of varying levels of physical activity, these data support the deleterious effects of time spent in sedentary activities. It would be beneficial for future research to attempt to disaggregate the separate contributions of physical activity and sedentary time on health outcomes related to maintaining independence in later life. 

In a recent study, light-intensity physical activity, measured objectively with an accelerometer, was significantly associated with better physical health, including lower extremity function, as well as other general health benefits among adults older than 65 years [20]. Although there are still considerations related to defining physical activity intensity using accelerometer cut-points in older adults, these data show health benefits, including higher PF associated with light activity. Avoiding sedentary activities may therefore confer important health benefits for some individuals even at activity levels below recommendations and supports the contention that some activity is better than none [6]. 

The present study also showed interesting trends related to sedentary time that may be partly explained by occupation and other socioeconomic variables. Contrary to other research that shows associations between obesity (and obesity-related chronic diseases) and low-income, less educated populations [28], these data tell a different story. Trends in education and income show higher percentages of the most highly educated and those with more income having greater amounts of sedentary time. This may be explained by occupation, number of work hours per day, and/or the ability to afford assistance with household chores, such as cleaning or yard work. 

There may also be occupational factors differentiating between sedentary hours and age. Between baseline and year 3, the greatest reductions in sedentary time occurred among the 50–69 year olds, which was approximately thirty-five minutes per day. Comparatively, women in the eldest age group reduced sedentary time by approximately twenty minutes per day. This trend among younger women may be related to entry into retirement, leading to less time spent doing seated tasks, such as computer work, and more time engaged in light, household, or leisure activities that may not always be captured as “physical activity,” especially when assessed by questionnaire. More active women also tended to not live alone, indicating that cohabitation is associated with less daily sedentary time and pointing to the potentially positive influence of social relationships in facilitating better health behaviors. 

These data are consistent with the hypothesis that participating in basic activities on a regular basis (e.g., simple household chores) could be a metabolic stimulus that helps to preserve ADLs and independence with aging. This may be especially true in the older subgroup of the elderly population, through preserving PF including skeletal muscle regulatory function. It is also important to note, however, that many self-reported physical activity questionnaires do not specifically ask about “light” activity, which may be quantified by the difference between moderate or vigorous activity and sedentary time. This research has important public health implications, given that declines in physical performance (strength, balance, and mobility) are associated with disability, morbidity, nursing home admissions, and other adverse consequences [29]—all of which confer substantial medical costs. 

Strengths of this study include the large sample size, inclusion of women diverse in race/ethnicity and socioeconomic status, more than ten years of prospective follow up on PF, and volume of data on potential confounders including the occurrence of incident disease. The findings are limited by not having objective measurements of sedentary time and not having a physical activity questionnaire that fully captured light intensity activity. This study was observational and does not establish a causal relationship between sedentary time and loss of physical function. Experimental studies are needed to test interventions that reduce sedentary time, and that explicate the role of relatively light intensity at lower than recommended volumes in preserving PF. 

The contribution of these data relates to both policy and clinical practice. First, expanding public health messages to reduce sedentary time *and* increase activity levels are likely to impart the broadest health benefits. Targeted messages to reduce time spent engaged in sedentary activities may have potential for impact when paired with current physical activity recommendations for older adults. Further, in conjunction with other findings [6, 30], these data provide a platform for clinicians to communicate with older patients about the benefits of general household and daily activities, irrespective of participation in a regular physical activity program. Although formal exercise participation is optimal, there appears to be a clear benefit of reducing sedentary activities among aging women that may confer extended duration of independent living and improved quality of life. 

## Figures and Tables

**Table 1:** Baseline characteristics of participants by total sedentary time, Women's Health Initiative Observational Study and Extension Study (WHI-OSES), (*N* = 61,609).

Characteristic	Total sedentary time (hours per day)^1^				*P* value^2^
	Q1 (≤6 hrs/day)	Q2 (>6–8 hrs/day)	Q3 (>8–11 hrs/day)	Q4 (>11 hrs/day)	
	*n* (%)	*n* (%)	*n* (%)	*n* (%)	
All	19364 (31.53%)	12316 (20.06%)	15958 (25.99%)	13769 (22.42%)	

Age group at screening, years					<.0001
50–59	6010 (31.04%)	3622 (29.41%)	5622 (35.23%)	6049 (43.93%)	
60–69	9213 (47.58%)	5927 (48.12%)	7097 (44.47%)	5623 (40.84%)	
70–79	4141 (21.39%)	2767 (22.47%)	3239 (20.30%)	2097 (15.23%)	

Ethnicity					<.0001
White	16507 (85.25%)	11103 (90.15%)	14355 (89.95%)	12301 (89.34%)	
Black	1387 (7.16%)	564 (4.58%)	743 (4.66%)	744 (5.40%)	
Hispanic	694 (3.58%)	264 (2.14%)	300 (1.88%)	248 (1.80%)	
American Indian	84 (0.43%)	36 (0.29%)	50 (0.31%)	34 (0.25%)	
Asian/Pacific Islander	410 (2.12%)	213 (1.73%)	326 (2.04%)	300 (2.18%)	
Unknown	282 (1.46%)	136 (1.10%)	184 (1.15%)	142 (1.03%)	

Income					<.0001
<$20,000	2513 (13.94%)	1327 (11.51%)	1671 (11.05%)	1387 (10.59%)	
$20,000–$34,999	3920 (21.74%)	2604 (22.59%)	3255 (21.52%)	2775 (21.19%)	
$35,000–$49,999	3724 (20.66%)	2408 (20.89%)	3100 (20.50%)	2670 (20.38%)	
$50,000–$74,999	3809 (21.13%)	2515 (21.82%)	3447 (22.79%)	2962 (22.61%)	
≥$75,000	4062 (22.53%)	2674 (23.20%)	3651 (24.14%)	3304 (25.23%)	

Education					<.0001
None-HS diploma	4040 (21.01%)	2217(18.11%)	2507 (15.84%)	2066 (15.12%)	
School after HS	7050 (36.67%)	4335(35.41%)	5557 (35.10%)	4685 (34.28%)	
College degree or higher	8135 (42.31%)	5689(46.47%)	7768 (49.07%)	6917 (50.61%)	

Physical activity					<.0001
Inactive (no reported recreational activity)	1971 (10.26%)	1250 (10.24%)	1815 (11.46%)	2243 (16.42%)	
<5 MET-hrs/week	3026 (15.75%)	2066 (16.92%)	2851 (18.01%)	2871 (21.02%)	
5-<12 MET-hrs/week	4251 (22.12%)	2830 (23.18%)	3942 (24.90%)	3382 (24.76%)	
≥12 MET-hrs/week	9967 (51.87%)	6064 (49.66%)	7223 (45.63%)	5162 (37.79%)	

Self-rate health status					<.0001
Excellent	4366 (22.62%)	2545 (20.72%)	3312 (20.79%)	2587 (18.83%)	
Very good	8399 (43.51%)	5552 (45.21%)	6984 (43.84%)	5764 (41.96%)	
Good	5324 (27.58%)	3525 (28.71%)	4624 (29.03%)	4188 (30.48%)	
Fair/poor	1214 (6.29%)	658 (5.36%)	1009 (6.33%)	1199 (8.73%)	

BMI					<.0001
<18.5	260 (1.36%)	135 (1.11%)	157 (0.99%)	112 (0.82%)	
18.5–24.9	8655 (45.20%)	5389 (44.27%)	6450 (40.87%)	5007 (36.83%)	
25–29.9	6646 (34.70%)	4112 (33.78%)	5520 (34.98%)	4464 (32.83%)	
>30.0	3589 (18.74%)	2536 (20.83%)	3655 (23.16%)	4013 (29.52%)	

Smoking					<.0001
Never	10296 (53.85%)	6323 (51.92%)	7874 (49.84%)	6425 (47.14%)	
Past	7873 (41.18%)	5258 (43.17%)	7127 (45.12%)	6380 (46.81%)	
Current	951 (4.97%)	598 (4.91%)	796 (5.04%)	825 (6.05%) %)	

Alcohol intake, per week					<.0001
0/past drinker	5456 (28.34%)	3078 (25.08%)	3793 (23.86%)	3381 (24.66%)	
<1	5966 (30.99%)	3851 (31.38%)	5180 (32.59%)	4732 (34.51%)	
1–14	6961 (36.16%)	4769 (38.85%)	6134 (38.59%)	4939 (36.02%)	
≥14	866 (4.50%)	576 (4.69%)	789 (4.96%)	661 (4.82%)	

Hormone use					<.0001
Never used	5485 (28.83%)	3452 (28.55%)	4304 (27.46%)	3526 (26.10%)	
Past user	3811 (20.03%)	2382 (19.70%)	3127 (19.95%)	2656 (19.66%)	
Current user	9729 (51.14%)	6257 (51.75%)	8245 (52.60%)	7326 (54.23%)	

Depressed mood					<.0001
0	5906 (31.00%)	3442 (28.26%)	4068 (25.70%)	3047 (22.36%)	
1-2	7137 (37.46%)	4840 (39.74%)	6183 (39.06%)	5191 (38.09%)	
3-4	3670 (19.26%)	2355 (19.33%)	3297 (20.83%)	2955 (21.68%)	
5+	2340 (12.28%)	1543 (12.67%)	2282 (14.42%)	2437 (17.88%)	

Living alone	4025 (20.90%)	2815 (22.96%)	4028 (25.34%)	3963 (28.92%)	<.0001

Activity of daily living disability (≥1 disability)	238 (1.27%)	139 (1.16%)	200 (1.29%)	281 (2.10%)	<.0001

History of CHD^3^	1098 (5.77%)	759 (6.24%)	958 (6.09%)	789 (5.82%)	0.2638

History of CHF	159 (0.84%)	99 (0.82%)	156 (1.00%)	126 (0.93%)	0.3204

History of stroke	217 (1.12%)	126 (1.02%)	159 (1.00%)	144 (1.05%)	0.6934

History of diabetes (use of pills or shots)	500 (2.59%)	295 (2.40%)	439 (2.75%)	442 (3.21%)	0.0003

Hypertensive (on medications for high blood pressure)	6826 (35.64%)	4340 (35.61%)	5594 (35.39%)	4715 (34.61%)	0.2261

History of arthritis	8672 (45.11%)	5852 (47.78%)	7590 (47.85%)	6577 (47.99%)	<.0001

History of cancer	2255 (11.73%)	1513 (12.36%)	1954 (12.32%)	1728 (12.61%)	0.0826

History of COPD^3^	533 (2.81%)	349 (2.89%)	507 (3.23%)	518 (3.83%)	<.0001

Number of falls in the previous 12 months					<.0001
None	13245 (69.14%)	8353 (68.47%)	10530 (66.64%)	8924 (65.44%)	
1 time	3741 (19.53%)	2436 (19.97%)	3254 (20.59%)	2867 (21.03%)	
2 times	1467 (7.66%)	928 (7.61%)	1372 (8.68%)	1212 (8.89%)	
3 or more times	703 (3.67%)	482 (3.95%)	646 (4.09%)	633 (4.64%)	

History of hip fracture at age ≥55 years	83 (0.54%)	70 (0.72%)	77 (0.63%)	67 (0.68%)	0.3034

Number of chronic diseases					<.0001
0	4515 (23.32%)	2699 (21.91%)	3406 (21.34%)	2934 (21.31%)	
1	6441 (33.26%)	4041 (32.81%)	5181 (32.47%)	4357 (31.64%)	
2	4661 (24.07%)	3065 (24.89%)	3955 (24.78%)	3391 (24.63%)	
3	2336 (12.06%)	1532 (12.44%)	2074 (13.00%)	1819 (13.21%)	
4	932 (4.81%)	656 (5.33%)	876 (5.49%)	850 (6.17%)	
5+	479 (2.47%)	323 (2.62%)	466 (2.92%)	418 (3.04%)	

^1^All WHI-OS participants who reported on sitting time and sleeping time at baseline were enrolled in the extension study and had filled out at least one Form 151 (*N* = 61,609). All the percentage calculations were based on number of participants who reported on the individual variable.

^2^ *P* value was based on chi-square test for the null hypothesis of no overall difference in the baseline variable among the 4 groups.

^3^Coronary heart disease (CHD) included MI, angina, CABG, and PTCA.

**Table 2:** Change in daily sitting time and total sedentary time between baseline and Year 3 of the OS, stratified by age.

Characteristic	All (*N*, mean (SD))	Age Categories (years)		
		50–59 (*N*, mean (SD))	60–69 (*N*, mean (SD))	70–79 (*N*, mean (SD))
Sitting time (hours/day)				
Baseline	61609, 7.47 (3.12)	21363, 8.07 (3.35)	27953, 7.23 (3.00)	12293, 6.97 (2.79)
Year 3	58111, 7.04 (2.93)	19888, 7.57 (3.20)	26541, 6.78 (2.77)	11682, 6.71 (2.67)
Change	58111, −0.43 (2.88)	19888, −0.50 (3.00)	26541, −0.45 (2.84)	11682, −0.27 (2.75)
*P* value for change^4^	<.0001	<.0001	<.0001	<.0001

Total sedentary time (hours/day)				
Baseline	61407, 8.63 (3.82)	21303, 9.14 (3.94)	27860, 8.42 (3.74)	12244, 8.21 (3.67)
Year 3	57565, 8.09 (3.44)	19697, 8.52 (3.62)	26308, 7.85 (3.33)	11560, 7.88 (3.30)
Change	57390, −0.54 (3.62)	19649, −0.61 (3.63)	26225, −0.58 (3.60)	11516, −0.33 (3.64)
*P* value for change^4^	<.0001	<.0001	<.0001	<.0001

^4^ *P* value was based on a 1-df *t*-test for the hypothesis of no significant change between baseline and year.

**Table 3:** Multivariate analysis of change in physical function score over time between WHI-OS baseline and WHI-OSES follow up and daily sitting time and total sedentary time^5^.

Key variable	Change in mean physical function score		
	Parameter estimate	95% CI	*P* value
Sitting time at baseline			<.0001
Q1 (≤4.5 hours/day)	1.00	(referent)	
Q2 (>4.5–6.5 hours/day)	−0.96	(−1.16, −0.76)	
Q3 (>6.5–8.5 hours/day)	−1.45	(−1.68, −1.22)	
Q4 (>8.5 hours/day)	−2.45	(−2.69, −2.22)	

Total sedentary time at baseline			<.0001
Q1 (≤6 hours/day)	1.00	(referent)	
Q2 (>6–8 hours/day)	−0.78	(−0.98, −0.57)	
Q3 (>8–11 hours/day)	−1.48	(−1.71, −1.25)	
Q4 (>11 hours/day)	−3.13	(−3.36, −2.89)	

^5^Results from linear mixed model of repeated measures assuming AR (1) covariance structure, adjusted for age race/ethnicity, education, physical activities, self-reported general health, BMI, smoking status, alcohol use, hormone use, depressed mood, living alone, activities of daily living, history of CHD, history of CHF, stroke, treated diabetes, arthritis, and history of COPD at baseline. Key variables were estimated in separated models and updated in the model with Year 3 data when available.
